# Editorial: Exploring the benefits of digital health technologies in diabetes management

**DOI:** 10.3389/fcdhc.2026.1843985

**Published:** 2026-06-16

**Authors:** Anouk Geraets, Artur Rydosz, Frank Snoek

**Affiliations:** 1 Department of Social Sciences, University of Luxembourg, Esch-sur-Alzette, Luxembourg; 2 Department of Electronics, AGH University of Krakow, Krakow, Poland; 3 Functional and Virtual 3D Medical Imaging Laboratory, Department of Diagnostic Imaging, University Hospital in Krakow, Krakow, Poland; 4 Department of Medical Psychology, Amsterdam University Medical Center, Vrije Universiteit Amsterdam, Amsterdam, Netherlands

**Keywords:** continuous glucose monitoring (CGM), diabetes management, digital health, mobile health (mHealth), technologies

We are pleased to introduce the Research Topic *“Exploring the Benefits of Digital Health Technologies in Diabetes Management*”. Digital health technologies (DHTs), including mobile health (mHealth) applications, continuous glucose monitoring (CGM), and artificial intelligence (AI)-driven tools, are reshaping diabetes care. These technologies enable real-time monitoring, personalized interventions, data-driven clinical decision-making, and patient engagement. There is growing evidence demonstrating that DHTs can improve glycemic outcomes, patient engagement, and support standard care models, at least on the short term (1, 2). However, there are questions around the long-term success, that depends on the integration into sustainable, patient-centered, and clinically embedded care pathways. This Research Topic brings together eleven articles: three systematic reviews, four original studies, one clinical trial, one methodological contribution, one opinion, and one brief research report, published across four Frontier Journals (Frontiers in Medicine, Frontiers in Digital Health, Frontiers in Clinical Diabetes and Healthcare, and Frontiers in Endocrinology). Collectively, these studies reflect the promise and the complexity of digital diabetes management strategies. The Research Topic highlights a shift in the field from technology-centered innovation towards implementation challenges involving patient engagement, workflow integration, equity, and responsible AI governance.

A central contribution of this collection lies in its mapping of the evolving research landscape in digital diabetes management. The systematic review by Zhu et al., which synthesizes 1,284 studies conducted across 101 countries between 2010 and 2024, identified AI as a rapidly expanding theme, particularly in predictive analytics and personalized care. This aligns with literature emphasizing the growing integration of machine learning into diabetes management for risk prediction and treatment optimization. In addition, a shift from technological innovation towards patient-centered approaches was observed. A trend echoed across several contributions in this Research Topic.

From intervention studies, improvements in glycemic control emerged as a recurring finding, although with notable variability across intervention types and populations. Roth et al. demonstrated that the ESYSTA digital application significantly improved HbA1c and patient-reported outcomes, such as well-being and diabetes-related distress, over six months. Similarly, Tang et al. reported improvements in HbA1c control rates, blood pressure, compliance rates, and user satisfaction among users of a mobile application. These findings are consistent with the systematic review by Stevens et al., which found greater HbA1c improvements in those who used DHTs.

However, the Research Topic also highlights conflicting findings that warrant critical reflection. Giovanazzi et al. found no difference in HbA1c reduction between users of the “TreC Diabete” platform and standard care after 12 months, despite high usability ratings. This discrepancy illustrates a broader challenge within digital diabetes care: improvements in usability do not necessarily translate into sustained improvements of clinical outcomes. Declining engagement over time, technical challenges, variability in digital literacy, and implementation barriers likely contribute to these mixed outcomes.

These findings underscore several methodological limitations that continue to affect the field. The studies included in this Research Topic vary in terms of intervention design, patient populations, follow-up duration, and outcome measures, limiting comparability. Many interventions are evaluated in controlled environments with short-term follow-up periods, leaving uncertainty regarding long-term adherence, sustainability, and scalability in routine clinical practice. Furthermore, the reliance on HbA1c may underestimate the effects of digital care on other important outcomes, such as quality of life, treatment burden, behavioral change, and patient empowerment.

The Research Topic also demonstrates the growing importance of continuous glucose monitoring technologies in the context of digital diabetes management. Guo et al. showed that ‘flash’ glucose monitoring, i.e. CGM requiring scanning, improved patient satisfaction, reduced pain, and enabled earlier detection of glucose abnormalities in post-renal-transplant patients compared with traditional blood glucose monitoring based on finger-pricking. In addition, Fleischer et al. demonstrated that CGM data can predict hypoglycemic events within a 40-minute window using so-called ensemble learning techniques. Together, these studies reinforce the role of CGM not only in daily monitoring of blood glucose excursions, but also in enabling predictive and preventive models of care. Beyond glycemic metrics, several studies emphasize psychosocial and behavioral dimensions of digital diabetes management. Hemetek et al. demonstrated that instant messaging services can facilitate peer interaction and knowledge exchange among individuals with type 2 diabetes. Similarly, Alzghaibi’s study found that wearable devices were perceived as valuable tools for monitoring and adherence support, although concerns about privacy, cost, and usability persist.

Collectively, these studies suggest that DHTs are most effective when they address clinical, behavioral, psychological, and social determinants of health. Although many interventions show promising short-term outcomes, maintaining adherence remains challenging, highlighting a broader implementation problem, requiring a critical analysis of barriers and enablers when aiming to bring the various digital tools into real-world care. Successful integration of DHTs into diabetes management requires at minimum interoperability and alignment with clinical workflows. Ultimately, the future of digital diabetes management will depend on integrated, patient-centered systems developed through collaboration among clinicians, researchers, engineers, policymakers, and people with lived experience. Equity considerations remain central in this context, as digital interventions may unintentionally widen disparities. In particular in populations with limited digital literacy, inadequate internet access, lower socioeconomic status, or language barriers. Recent literature emphasizes that equitable implementation strategies must accompany technological innovation to avoid reinforcing existing health inequities (2–4). Accordingly, future research should prioritize accessibility, cultural sensitivity, and inclusive recruitment.

The role of AI and machine learning represents one of the most transformative yet challenging dimensions of digital diabetes management. Corrao et al. highlight the potential of AI-driven systems to support early detection of complications, personalized treatment pathways, and advanced monitoring, while Lazarus et al. emphasize the need for integrated, multi-source digital monitoring systems in diabetic foot ulcer management. However, the rapid expansion of AI also raises important clinical, ethical, and regulatory concerns. Key challenges include data quality, algorithmic bias, limited transparency, insufficient prospective clinical validation, and poor interoperability with electronic health records and existing healthcare infrastructure. These limitations may hinder clinician and patient trust, adoption, and equitable implementation, particularly among underrepresented populations. As AI becomes increasingly integrated into diabetes care, robust governance frameworks that address cybersecurity, data privacy, regulatory oversight, and accountability will be essential to ensure the safe, transparent, and equitable deployment of these technologies (5–7).

Several priorities for future research emerge from this Research Topic. First, there is a need to move beyond HbA1c as the sole primary outcome and incorporate more nuanced measures such as time-in-range, glycemic variability, and hypoglycemic events, along with patient-reported outcomes (8). Second, long-term studies are required to assess sustainability and real-world effectiveness (9). Third, implementation should be incorporated more systematically into digital health research to better understand and promote adoption, engagement, scalability, and health system integration (10, 11). Finally, future studies should prioritize ethical AI development, equitable deployment strategies, and transparent governance structures (5–7).

In conclusion, this Research Topic provides a timely, comprehensive and multifaceted overview of both the opportunities and limitations of DHTs in diabetes management. Digital technologies are rapidly evolving and can potentially improve monitoring, support self-management, and facilitate more personalized approaches to care. Yet, persistent challenges remain related to engagement sustainability, methodology, implementation, equity, and AI governance. Addressing these issues is needed to ensure that digital diabetes management evolves into a clinically effective, equitable, and sustainable component of future diabetes care.
